# Facial Melanosis: A Clinico-Dermoscopic Evaluation and Quality of Life Assessment

**DOI:** 10.7759/cureus.105077

**Published:** 2026-03-11

**Authors:** Anuradha Yadav, Arushi Nanda, Sanjeev Gupta, Aneet Mahendra, Aditi Dabhra

**Affiliations:** 1 Dermatology, Yadav Diagnostic Centre and Skin Hospital, Narnaul, IND; 2 Dermatology, Maharishi Markandeshwar Institute of Medical Science and Research, Ambala, IND

**Keywords:** diagnostic dermoscopy, facial hyperpigmentation, lichen planus pigmentosus, melasma and facial melanosis, quality of life

## Abstract

Background: Facial melanoses (FM) represent a heterogeneous group of pigmentary disorders characterized by altered facial pigmentation, including melasma, lichen planus pigmentosus (LPP), Riehl’s melanosis, erythema dyschromicum perstans, and others. These conditions constitute a major proportion of dermatology outpatient visits and, despite being largely asymptomatic, exert a substantial negative impact on quality of life (QoL) due to cosmetic disfigurement. The etiology of many forms of FM remains unclear, and studies correlating clinical, dermatoscopic, and QoL parameters are limited.

Study design: A prospective observational study.

Primary objective: To evaluate the change in QoL in patients with FM after three months of diagnosis-based treatment, using the Dermatology Life Quality Index (DLQI) and Melasma Quality of Life Scale (MELASQoL) scores.

Secondary objectives: To describe the clinical profile of patients with FM and to identify the etiological spectrum of the condition in the study population. The study also aimed to assess Wood’s lamp findings and dermoscopic patterns of the lesions and to document histopathological findings in selected cases, correlating them with the clinical and dermoscopic features wherever possible.

Materials and methods: A prospective observational study was conducted among 50 patients aged 18-60 years with FM involving more than 50% of the facial surface area, attending a tertiary care hospital. Detailed clinical evaluation, Wood’s lamp examination, and dermatoscopic assessment were performed in all patients. QoL was assessed using the DLQI and MELASQoL at baseline and after three months of treatment. Histopathology was undertaken where clinically indicated. Statistical analysis was performed using SPSS v28 (IBM Corp., Armonk, NY).

Results: The mean age of participants was 35.7 years; 38 patients (76%) were female, yielding a female-to-male ratio of 3.16:1. LPP was the most common diagnosis, found in 26 (52%) patients, followed by melasma, found in nine (18%) patients. Dermatoscopy most frequently demonstrated an accentuated reticular pattern in 21 (42%) patients and globular pigmentation in 18 (35%) patients, with gray granules correlating with dermal pigmentation in LPP. The mean DLQI score improved from 7.86±6.64 at baseline to 4.78±6.12 at three months, while the mean MELASQoL score decreased from 36.04±21.03 to 26.36±17.42 (p<0.001 for both). Hypothyroidism was the most common associated comorbidity, observed in five (10%) patients.

Limitations: Histopathological confirmation was done in limited cases, and the single-center design with a modest sample size may limit generalizability.

Conclusion: FM significantly impairs QoL, particularly among young women. LPP emerged as the predominant cause in our study. Dermatoscopy is a valuable non-invasive tool for assessing pigment depth and guiding management. DLQI and MELASQoL are effective instruments for monitoring therapeutic outcomes. Early diagnosis, appropriate counseling, and multimodal therapy are essential to improve patient well-being.

## Introduction

Facial melanosis (FM) can significantly affect quality of life (QoL). Some patients feel so embarrassed or self-conscious that it hampers their day-to-day routines and normal activities. In severe cases, patients may experience social withdrawal and avoidance behaviors, significantly affecting psychological well-being. Young females, especially those in the marriageable age group, are the most severely affected [1,2].

Diagnosis is generally based on a detailed history and clinical features, with or without histopathology, dermatoscopy, or Wood's lamp examination. In cases where a biopsy is not possible or is refused by the patient, dermatoscopy proves to be very useful. It can also be used to monitor the patient’s response to treatment.

Different modalities of treatment are available for FM, but the outcome is still far from ideal. Therefore, combination therapy is often employed to achieve satisfactory outcomes [3].

Adequate counseling of the patient is vital for management, as it takes months to years for effective treatment, requiring multiple visits to the hospital and regular use of medications. Since improvement is gradual and there are no instant results within days, patients are sometimes lost to follow-up because some want instant results, while others simply cannot afford the treatment.

## Materials and methods

Study design

This was a prospective observational study.

Primary objective

To evaluate the change in QoL in patients with FM after three months of diagnosis-based treatment using the Dermatology Life Quality Index (DLQI) and the Melasma Quality of Life Scale (MELASQoL) scores.

Secondary objectives

To describe the clinical profile of patients with FM and to identify the etiological spectrum of the condition in the study population. The study also aimed to assess Wood’s lamp findings and dermoscopic patterns of the lesions and to document histopathological findings in selected cases, correlating them with the clinical and dermoscopic features wherever possible.

Study area

The present study was conducted in the Dermatology Outpatient Department of Maharishi Markandeshwar Institute of Medical Sciences and Research, Mullana, Ambala, over a period of one and a half years, starting from December 20, 2018, until June 19, 2020. A total of 62 patients with clinically diagnosed FM were initially screened according to predefined selection criteria. Out of these, six patients declined to participate, and six were lost to follow-up. Consequently, 50 patients who completed all study visits were included in the final analysis.

Inclusion criteria

Patients of either sex aged 18-60 years, presenting to the dermatology outpatient department with clinical features of FM involving more than 50% of the facial area, were included in the study. The extent of facial involvement was assessed by clinical estimation during dermatological examination, based on the proportion of visible facial surface area affected by hyperpigmentation across standard anatomical regions of the face.

Exclusion criteria

Patients with involvement of less than 50% of the facial area, pregnant or lactating women, patients with congenital pigmentary disorders of the face (e.g., congenital dermal melanocytosis), and patients with any active cutaneous infection at the local site were excluded from the study.

Analysis of sample size

The sample size for this study was calculated using the standard formula for estimating a population proportion. In this formula, Z represented the standard normal value corresponding to the desired confidence level (a value of 1.96 was used for a 95% CI). The term p denoted the expected proportion of the outcome in the population, and d represented the allowable margin of error (absolute precision).

Strategy

Written informed consent was obtained from all participants prior to enrollment. After confirmation of eligibility according to the inclusion criteria, a detailed history was recorded, including presenting complaints, duration of illness, cosmetic use, previous treatment history, and associated comorbidities. A comprehensive dermatological examination was performed, and clinical photographs were obtained for documentation. QoL was assessed at baseline using the DLQI [4] and the MELASQoL [5]. Baseline laboratory investigations, including hemoglobin, thyroid function tests, random blood sugar, and peripheral blood film examination, were carried out in all patients. Wood’s lamp examination was performed to evaluate the depth of pigmentation. Dermoscopic examination was performed using a Dino-Lite video dermoscope (AM4113ZT) under polarized light mode, and images were evaluated to identify pigmentary patterns such as reticular networks, globules, and granular pigmentation. A 4 mm punch biopsy from representative lesions was obtained for histopathological examination, wherever clinically indicated and with patient consent. The final diagnosis was established based on clinical evaluation supported by dermoscopic and Wood’s lamp findings, and histopathological confirmation in selected cases where the diagnosis was uncertain. Treatment was individualized according to the final clinical diagnosis and disease severity. Patients with melasma were treated with strict photoprotection and topical depigmenting agents such as hydroquinone-based combinations. Patients with lichen planus pigmentosus (LPP) received anti-inflammatory therapy, including topical corticosteroids or topical calcineurin inhibitors, along with photoprotection. Cases of topical steroid-damaged facies were managed with gradual withdrawal of topical steroids and barrier-repair therapy. Drug-induced pigmentation was addressed by discontinuation of the offending drug where feasible. Patients with airborne contact dermatitis (ABCD)-induced pigmentation were treated with allergen avoidance and anti-inflammatory therapy. All patients were counseled regarding sun avoidance and regular use of broad-spectrum sunscreen. All patients were followed up at three months and were reassessed through clinical examination, photographic documentation, dermatoscopic evaluation, and repeat QoL assessment using DLQI [4] and MELASQoL [5].

Analysis of data

All tools and scoring systems used in this study are freely available for academic use. Statistical analysis was performed using IBM SPSS Statistics for Windows, Version 28.0 with ID-332076 (IBM Corp., Armonk, New York, United States), which was used under an institutional license. Normality of the continuous variables (DLQI and MELASQoL scores) was assessed prior to inferential analysis using the Shapiro-Wilk test. As the data were not normally distributed, a nonparametric test (Wilcoxon signed-rank test) was used for comparison of pre- and post-treatment scores. Continuous variables were summarized as mean±standard deviation, while categorical variables were expressed as frequencies and percentages. No proprietary clinical questionnaires or licensed scoring instruments were used in this study.

Ethical consideration

Institutional Ethics Committee approval (IEC-256) was obtained prior to study initiation. All participants were enrolled after providing a detailed explanation of the study, and written informed consent was obtained. Confidentiality of patient information was ensured throughout the study.

## Results

The study included 50 patients with FM. The largest proportion of patients, 19 (38%), belonged to the 31-40 year age group, with a mean age at presentation of 35.7 years. A female preponderance was observed, with a female-to-male ratio of 3.16:1.

The mean duration before seeking medical consultation was three years. The majority of patients, 16 (32%), reported facial pigmentation of 1-2 years' duration, followed by 12 (24%) patients with a duration of less than one year. Seven (14%) patients had pigmentation persisting for more than five years. Pruritus was present in 17 (34%) patients, while 33 (66%) were asymptomatic. The overall duration of melanosis ranged from 15 days to 15 years.

The majority of patients, 42 (84%), were engaged in indoor occupations, while eight (16%) had outdoor occupations. Seasonal exacerbation of pigmentation during summer months was reported by six (12%) patients, and 21 (42%) had a history of photosensitivity. A positive family history of FM was present in three (6%) patients, all of whom were diagnosed with melasma. Associated systemic comorbidities were noted in 15 (30%) patients, with hypothyroidism being the most common, followed by diabetes mellitus, Addison's disease, and hyperthyroidism.

A history of prior treatment with topical or oral medications was noted in 36 (72%) patients. All of these patients had used topical preparations, while 30 (60%) had also received oral medications. Cosmetic product use was reported by 20 (40%) patients, of whom 19 were females. Overall, 11 distinct categories of FM were identified (Table 1).

**Table 1:** Types of FM *SPSS software version 28 (IBM Corp., Armonk, New York, United States was used to analyze the data. LPP, Lichen planus pigmentosus; FM, facial melanosis

Diagnosis		Male, n (%)	Female, n (%)	Total, n (%)
LPP		6 (23.1%)	20 (76.9%)	26 (52%)
Melasma		0 (0%)	9 (100%)	9 (18%)
Drug-induced pigmentation		1 (33.3%)	2 (66.7%)	3 (6%)
Topical steroid damaged facies		0 (0%)	3 (100%)	3 (6%)
Airborne contact dermatitis-induced pigmentation		1 (50%)	1 (50%)	2 (4%)
Occupational pigmentation		2 (100%)	0 (0%)	2 (4%)
Addison’s disease		1 (100%)	0 (0%)	1 (2%)
Nevus of Ota		0 (0%)	1 (100%)	1 (2%)
Pityriasis lichenoides chronicus		0 (0%)	1 (100%)	1 (2%)
Post-inflammatory hyperpigmentation		0 (0%)	1 (100%)	1 (2%)
Riehl’s melanosis		1 (100%)	0 (0%)	1 (2%)
Total		12 (24%)	38 (76%)	50 (100%)
Fisher’s exact test*	x^2^	18.3		
	p-value	0.028		

The most common diagnosis was LPP, observed in 26 (52%) patients, followed by melasma in nine (18%) patients. Drug-induced pigmentation and topical steroid-dependent face (TSDF) were each reported in three (6%) patients. Occupational hyperpigmentation and ABCD-induced pigmentation were noted in two (4%) patients each. Riehl’s melanosis, pityriasis lichenoides chronica (PLC), post-inflammatory hyperpigmentation (PIH), acquired nevus of Ota, and Addisonian pigmentation were each identified in one (2%) patient.

On Wood’s lamp examination, nine (18%) patients demonstrated accentuation of pigmentation, whereas 41 (82%) showed no accentuation. All patients demonstrating accentuation under Wood’s lamp examination were diagnosed with melasma.

On dermatoscopic evaluation, the accentuated reticular pattern was the most common finding, observed in 21 (42%) patients, followed by a globular pattern in 18 (35%) patients. Background pigmentation was light brown in 17 (33%) patients and dark brown in 14 (27%) patients. Gray pigmentation, suggestive of dermal melanin, was noted in 18 (35%) patients. Regarding pigment distribution, granular clumps were seen in 14 (27%) patients, a combination of granules and globules in 10 (20%) patients, and globules alone in three (5%) patients (Table 2).

**Table 2:** Dermatoscopic findings in study participants

Parameter	Finding	Number of patients (n)	Percentage
Dermatoscopic pattern	Accentuated reticular pattern	21	42%
	Globular pattern	18	35%
Background pigmentation	Light brown	17	33%
	Dark brown	14	27%
Additional pigment color	Grey pigmentation	18	35%
	Dark brown pigmentation	17	34%
Pigment distribution	Granular clumps	14	27%
	Granules + globules	10	20%
	Globules alone	3	5%

**Figure 1:**
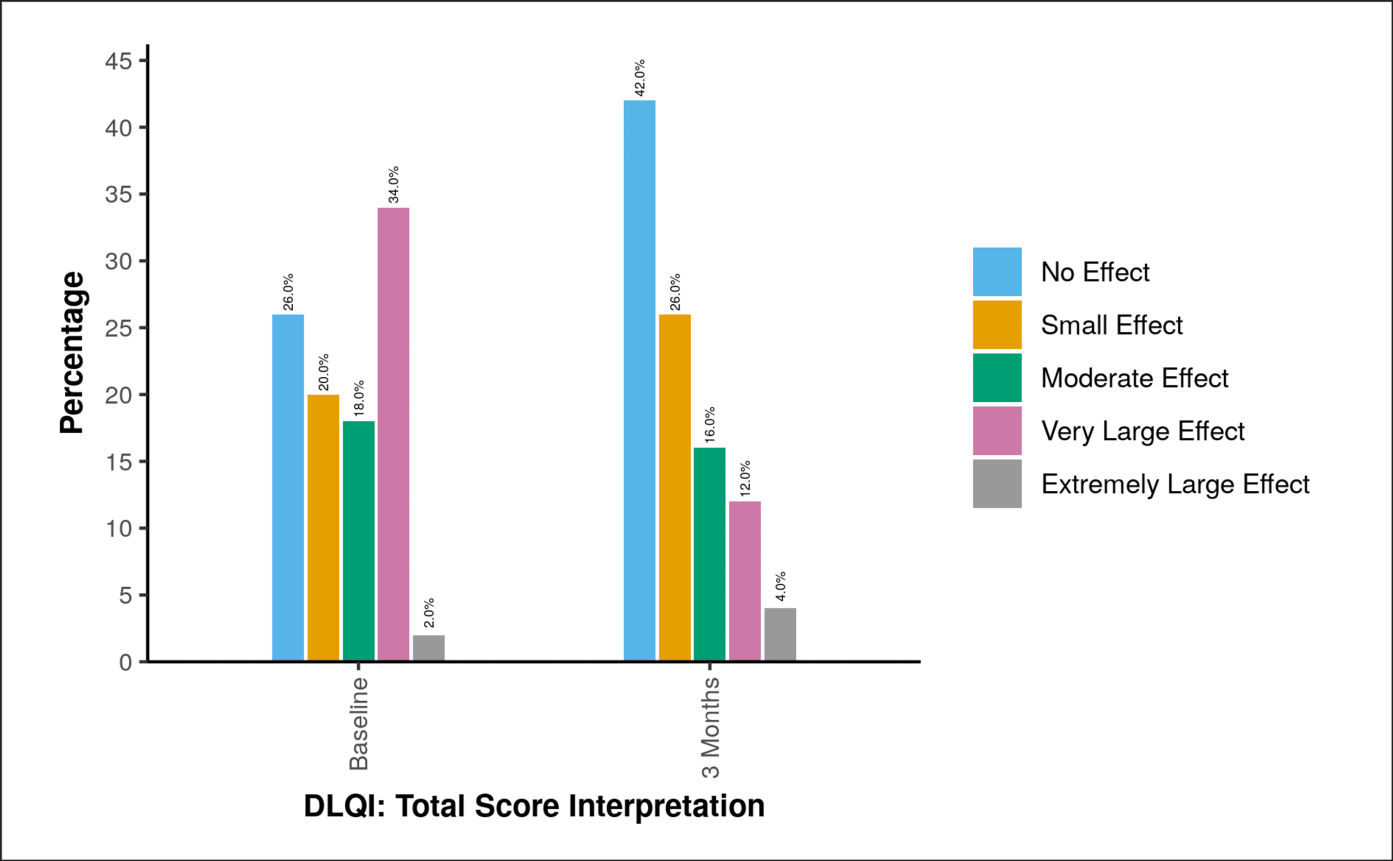
Graph showing change in DLQI: total score interpretation over time Change in DLQI [4]: Total score interpretation over time. DLQI, Dermatology Life Quality Index

At presentation, the majority of patients, 17 (34%), reported a “very large effect” on their QoL, while 13 (26%) had “no effect” as per DLQI categorization. After three months of treatment, a marked improvement was observed, with 21 (42%) patients reporting “no effect” on their QoL (Figure 1).

The mean DLQI [4] score showed a significant reduction from 7.86±6.64 at baseline to 4.78±6.12 after three months of treatment. Similarly, the mean MELASQoL [5] score improved from 36.04±21.03 at baseline to 26.36±17.42 at three months. Using the Wilcoxon signed-rank test, the improvement in both indices was found to be statistically significant (DLQI: V=860.5, p<0.001; MELASQoL: V=752.0, p<0.001) (Table 3).

**Table 3:** Mean scores of DLQI and MELASQoL, pre- and post-treatment Mean scores of DLQI [4] and MELASQoL [5], pre- and post-treatment (Wilcoxon test statistics, SPSS software version 28, IBM Corp., Armonk, New York, United States).

Score	Pre-treatment (mean±SD)	After three months (mean±SD)	Wilcoxon test statistic (V)	P-value
DLQI	7.86±6.64	4.78±6.12	860.5	<0.001
MELASQoL	36.04±21.03	26.36±17.42	752.0	<0.001

Among the 50 patients, nine (18%) had low hemoglobin levels (<12 g/dL). Raised thyroid-stimulating hormone levels (>5 mIU/L) were observed in five (10%) patients, and two (4%) patients had elevated random blood sugar levels (>140 mg/dL). Peripheral blood film examination was normal in all patients.

## Discussion

FM has long been recognized as a challenging condition for dermatologists, primarily because treatment often extends over several months, which contrasts with patients’ expectations of rapid improvement. Any alteration in skin color, particularly on the face, is immediately noticeable and frequently becomes a source of significant emotional stress. Such visible pigmentation changes contribute to cosmetic disfigurement and considerable psychological discomfort, adversely affecting self-esteem and social interactions.

Sociocultural influences, including media portrayals favoring lighter skin tones, may contribute to increased concern regarding complexion and have further intensified concerns regarding complexion in the Indian population. This heightened preference for fair skin often drives individuals to use unregulated or inappropriate skin-lightening measures, which may damage the cutaneous barrier and paradoxically precipitate or worsen melanosis.

Females are generally more concerned about cosmetic appearance and are therefore more likely to seek medical consultation for aesthetic problems than males. A similar trend was observed in our study, in which 38 (76%) out of 50 patients were females, and 12 (24%) were males, yielding a female-to-male ratio of 3.16:1. This female predominance may also be explained by the fact that LPP, the most common diagnosis in our study, is known to occur more frequently in women. Comparable female preponderance has been documented in previous studies on FM, particularly in cohorts of melasma and LPP, where women constituted the majority of affected individuals [6,7].

Facial hyperpigmentation is predominantly observed in younger adults, as several implicated factors, such as pregnancy, use of oral contraceptive pills, cosmetics, and certain drugs, are more prevalent in this age group. In our study, the largest proportion of patients, 19 (38%), belonged to the 31-40 years age group, followed by 14 (28%) patients in the 21-30 years age group; these findings are consistent with previous reports [6].

Excessive sun exposure is considered a significant contributing factor in the development of FM, particularly among individuals with darker skin types. In our study, housewives engaged in a combination of indoor and outdoor activities constituted the most commonly affected group. Seasonal exacerbation of pigmentation during summer months, suggestive of photosensitivity, was reported in six (12%) patients. A positive family history was documented in three (6%) patients, all of whom were diagnosed with melasma, highlighting the role of genetic predisposition in this disorder.

Facial hyperpigmentation often drives patients to use a variety of fairness creams and over-the-counter products in pursuit of a lighter complexion, largely influenced by media advertisements promoting instant skin brightening. Several potent agents, such as hydroquinone and topical corticosteroids, which ideally should be dispensed only on prescription, are easily accessible without medical supervision. Indiscriminate and prolonged use of these preparations can disrupt the skin barrier, induce irritation, rebound pigmentation and, in some cases, lead to conditions such as TSDF. In the present study, a history of cosmetic use was reported by 20 (40%) patients, underscoring the significant role of unsupervised topical applications in the development and aggravation of facial hyperpigmentation.

Several systemic disorders have been reported in association with FM, including thyroid dysfunction, diabetes mellitus, and hypertension. In the present study, 15 (30%) patients had at least one associated systemic illness, with hypothyroidism being the most frequently observed comorbidity, a finding consistent with previous studies [8]. Hypothyroidism was identified in six (11.5%) patients with LPP and in 17 (33.3%) patients with melasma, further supporting the possible role of endocrine dysfunction in the pathogenesis and persistence of facial hyperpigmentation.

FM encompasses a heterogeneous group of disorders such as melasma, LPP, erythema dyschromicum perstans (EDP), and Riehl’s melanosis. In most studies, melasma is reported as the most frequent cause of facial hyperpigmentation; however, in the present study, melasma was the second most common diagnosis, observed in nine (18%) patients (Table 1). The leading cause of FM in our study was LPP, identified in 26 (52%) patients (Table 1). This variation may be attributed to the study inclusion criteria, which required involvement of more than 50% of the facial area; melasma often affects relatively smaller and more localized regions, and such patients were therefore not included.

LPP is regarded as a pigmented variant of lichen planus and may occasionally be associated with classical lichen planus lesions at other sites. In the present study, coexistent typical lichen planus lesions were noted in only one patient (3.84%) among those diagnosed with LPP, suggesting that isolated facial involvement is more common in this population.

Dermatoscopy is a noninvasive, in vivo diagnostic technique that enables visualization of subtle structural and pigmentary patterns of the skin that are not appreciable to the unaided eye. In many situations, it serves as a valuable tool for diagnosis and follow-up, and may even obviate the need for invasive skin biopsy. In our study, dermoscopic evaluation of LPP lesions revealed pigmentation predominantly in the form of granules and globules in 15 (30%) patients and as globules alone in 18 (35%) patients. Chamli et al. [9] demonstrated a strong correlation between dermoscopic gray dots and globules and histological pigment incontinence in LPP, findings that are in concordance with the patterns observed in our study.

The pigment distribution in LPP was characteristically nonuniform, with blunting around hair follicles and sweat gland openings. Dermatoscopy was also helpful in differentiating LPP from erythema dyschromicum perstans, which typically shows a more uniform color distribution with an active erythematous border. In contrast, melasma in our patients exhibited follicular sparing on dermoscopy, a feature similar to that described by Yalamanchili et al. [6], who reported a reticular pigment network with perifollicular sparing as a hallmark dermoscopic sign of melasma.

Wood’s lamp examination is a useful bedside tool for assessing the depth of pigmentation. Epidermal pigmentation typically shows accentuation under Wood’s light, whereas dermal pigmentation remains unchanged. In the present study, accentuation of lesions was observed in nine (18%) patients, while 41 (82%) showed no accentuation. Notably, all patients demonstrating accentuation were diagnosed with melasma, supporting its predominantly epidermal component.

Facial hyperpigmentation has occasionally been reported as an early manifestation of vitamin B12 deficiency. However, due to financial constraints, serum vitamin B12 levels could not be assessed in our patients; instead, peripheral blood film examination was performed as a surrogate indicator, and it was normal in all 50 patients. Evaluation of other systemic parameters revealed hypothyroidism in seven (14%) patients, low hemoglobin in nine (18%) patients, and raised random blood sugar in two (4%) patients, indicating the presence of potentially contributory comorbidities. These patients had partial improvement on treatment.

FM can exert a profound impact on an individual’s QoL owing to its visible and often disfiguring nature. The effect of the disease on psychological well-being, interpersonal relationships, and occupational functioning has increasingly become an important aspect of patient assessment. The mean baseline DLQI score of 7.86 corresponds to a moderate effect on QoL according to the validated DLQI banding categories, although 34% of patients individually experienced a very large effect. Furthermore, the observed reduction in mean DLQI score from 7.86 to 4.78 represents a change that is not only statistically significant but also clinically meaningful, as a reduction of approximately four points is generally considered the minimal clinically important difference.

After three months of therapy, a statistically significant improvement was observed in both DLQI [4] and MELASQoL [5] scores (p<0.001), indicating a meaningful enhancement in patient-reported outcomes. Comparable findings have been documented in previous studies; Gupta et al. [7] reported greater QoL impairment in LPP compared to melasma, while Reza Almasi Ghale et al. [10] demonstrated a strong correlation between disease severity and DLQI scores in patients with melasma. These observations underscore the importance of addressing the psychosocial dimensions of FM alongside clinical management.

Skin pigmentation can be induced by a wide range of medications, including oral contraceptive pills, amiodarone, antimalarials, clofazimine, hydroxyurea, anticonvulsants, and tetracyclines. Drug-induced pigmentation may show partial resolution after discontinuation of the offending agent; however, in a subset of patients, hyperpigmentation can persist for prolonged periods despite long-term withdrawal. In the present study, drug-induced pigmentation was identified in three (6%) patients. One of these patients had been receiving hydroxyurea 500 mg daily for the preceding four months for the management of polycythemia vera, which was considered the likely etiological factor.

Hyperpigmentation in Addison’s disease is characteristically diffuse and most pronounced over sun-exposed areas, pressure points, and mucosal surfaces. The underlying mechanism is attributed to increased secretion of melanotrophic hormones, particularly adrenocorticotropic hormone, by the pituitary gland in response to adrenal insufficiency. In our study, the patient diagnosed with Addison’s disease presented with generalized pigmentation involving the entire body, including the oral mucosa, with a history of progression over the past 15 years. The patient showed partial improvement after treatment.

Dermatoscopic features in FM closely mirror the level and distribution of melanin deposition observed on histopathology. Gray to slate-blue pigmentation and granular structures, predominantly seen in LPP and drug-induced pigmentation, correspond to dermal melanophages resulting from pigment incontinence, where the Tyndall effect over dermal melanin produces characteristic gray hues. In contrast, the light-to-dark brown pseudoreticular or globular patterns observed mainly in melasma reflect epidermal basal hypermelanosis and elongation of rete ridges, with minimal dermal involvement.

Perifollicular gray dots and an accentuated reticular network, considered hallmark dermatoscopic features of LPP, correlate with lichenoid interface dermatitis and perifollicular accumulation of melanophages on histology. Mixed brown-gray patterns, as noted in TSDF and drug-induced pigmentation, indicate combined epidermal and dermal melanin deposition, suggesting chronicity and a potentially poorer response to topical depigmenting therapies. Dermatoscopy serves as a valuable noninvasive adjunct that may correlate closely with histopathological findings, facilitating differentiation between epidermal and dermal pigmentation, guiding therapeutic decisions, and helping to avoid skin biopsy in many patients [11,12].

A 4 mm punch biopsy from representative lesions was obtained for histopathological examination in a limited number of selected cases where the clinical and dermatoscopic findings were inconclusive or overlapping, and histological confirmation was considered necessary. Among patients with LPP, biopsy was undertaken when clinically indicated and typically demonstrated features of interface dermatitis, including basal layer vacuolar degeneration and prominent pigment incontinence. A biopsy was also performed in the case of Riehl’s melanosis, which revealed patchy basal cell vacuolar alteration with mild spongiosis, along with dermal pigment incontinence and a perivascular lymphocytic infiltrate.

Although the diagnosis of FM is largely based on clinical evaluation, histopathology serves as an important adjunct, particularly in atypical or overlapping presentations. Previous studies have examined various aspects of facial hyperpigmentation, such as clinical characteristics, dermatoscopic patterns, histopathological features, and QoL, largely as isolated parameters. Our study aimed to assess these dimensions in an integrated manner to provide a more comprehensive understanding of the disorder (Table 4).

**Table 4:** Summary of various studies on FM LPP, lichen planus pigmentosus; QoL, quality of life; FM, facial melanosis; DLQI, Dermatology Life Quality Index; MELASQoL, Melasma Quality of Life Scale; MASI, Melasma Area and Severity Index

S.no	Year	No. of patients	Author	Clinical	Dermoscopy	Biopsy	QoL	Blood Inv.	Most common diagnosis	Remarks
1	2015	140	Yalamanchili et al. [6]	Yes	Yes	No	Yes (MELASQoL)	No	Melasma	The malar pattern was most common, and dermoscopy typically showed a reticular pigment network with perifollicular sparing. The mean MELASQoL score was high
2	2021	246	Gupta et al. [7]	Yes	No	No	Yes (DLQI-modified)	No	LPP and Melasma	LPP was associated with significantly poorer QoL than melasma, with higher modified DLQI scores.
3	2017	30	Bhat et al. [9]	Yes	No	Yes	No	Yes	LPP	Clinical evaluation showed female preponderance and photo-aggravation in many cases. Histopathology demonstrated epidermal atrophy, melanin incontinence, and inflammatory infiltrate. Significant correlations among CD4+, CD8+, CD45RO, and CD68 cell markers
4	2025	111	Reza Almasi Ghale et al. [10]	Yes	No	No	Yes (DLQI)	No	Melasma	Higher MASI scores correlated strongly with poorer QoL (mean DLQI ≈ 6.16).
5	2024	23	Chamli et al. [13]	Yes	Yes	Yes	No	No	LPP	Dermoscopy predominantly showed dots/globules with exaggerated pseudo-reticular pattern and consistent follicular sparing. Histopathology correlated with pigment incontinence, and dermoscopic blotches were associated with chronic disease.
6	2020	238	Raveendra et al. [14]	Yes	No	No	Yes (Skindex-16)	No	Melasma	Melasma was the most common diagnosis (73%). QoL showed moderate impairment overall, but the severity and extent of pigmentation did not correlate significantly with QoL scores, suggesting even mild pigmentation impacts patients psychologically. Females had a higher emotional burden compared to males.
7	2018	155	Pollo et al. [15]	Yes	No	No	Yes (MELASQoL)	No	Melasma	Higher clinical severity (MASI) and greater socioeconomic burden were associated with poorer QoL measured by MELASQoL. Factors such as lower education and longer disease duration significantly worsened QoL
8	2023	150	Mpofana et al. [16]	Yes	No	No	Yes (MELASQoL)	No	Melasma	Higher MASI scores, cheek involvement, education level, and menopausal status were significant predictors of poorer MELASQoL.
9	2020	50	Present study	Yes	Yes	Yes	Yes	Yes	LPP	LPP as the most common diagnosis (52%), followed by melasma (18%). Dermoscopy most frequently revealed an accentuated reticular pattern, and QoL improved significantly after three months with a reduction in DLQI (7.86→4.78) and MELASQoL (36.04→26.36) scores (p<0.001).

Treatment was instituted according to the final clinical diagnosis and standard therapeutic protocols. Treatment was individualized according to the final clinical diagnosis and disease severity. All patients received counseling regarding sun avoidance measures and regular use of broad-spectrum sunscreen. Treatment adherence was assessed during follow-up visits by patient interview.

This study evaluated the clinico-dermatoscopic characteristics and QoL in patients with facial hyperpigmentation, along with the impact of treatment on patient-reported outcomes. An attempt was made to comprehensively correlate clinical features, dermatoscopic patterns, and QoL measures, as only a limited number of studies have examined these parameters together in an integrated manner in FM. Limitations of this study include that the inclusion of a heterogeneous group of FM disorders with relatively small numbers in individual diagnostic subgroups precluded meaningful subgroup analyses, such as separate analyses for LPP and melasma. Also, the study was conducted at a single tertiary care center with a modest sample size, which may limit the generalizability of the findings and introduce the possibility of selection bias. The follow-up period was limited to three months, which may not fully capture the long-term clinical course or sustained QoL changes associated with FM. Furthermore, as this was a prospective observational study without a control group, causal relationships between treatment interventions and improvements in QoL scores cannot be definitively established. Finally, given the exploratory nature of the study and the heterogeneity of diagnoses, the statistical analysis primarily relied on nonparametric hypothesis testing to assess changes over time, and formal reporting of effect sizes and confidence intervals for paired differences was not performed.

## Conclusions

FM represents a heterogeneous group of disorders with significant cosmetic and psychosocial impact. In our study, LPP emerged as the most common cause, followed by melasma. Dermatoscopy proved to be a valuable, noninvasive tool that closely correlated with histopathological findings and aided in differentiating epidermal from dermal pigmentation, thereby guiding diagnosis and management while reducing the need for facial biopsy. QoL was substantially impaired at baseline, underscoring the psychological burden associated with facial pigmentation. Significant improvement in DLQI and MELASQoL scores following treatment highlights the importance of timely and appropriate therapeutic intervention. The frequent association with systemic comorbidities, particularly hypothyroidism, further emphasizes the need for a holistic approach to evaluation. By integrating clinical, dermatoscopic, histopathological, and QoL parameters, this study attempts to provide a more comprehensive understanding of FM. Larger, multicentric studies with longer follow-up are recommended to further elucidate disease behavior and optimize management strategies.
